# Utilizing students’ experiences and opinions of feedback during problem based learning tutorials to develop a facilitator feedback guide: an exploratory qualitative study

**DOI:** 10.1186/s12909-015-0507-y

**Published:** 2016-01-11

**Authors:** Aloysius Gonzaga Mubuuke, Alwyn J. N. Louw, Susan Van Schalkwyk

**Affiliations:** School of Medicine, College of Health Sciences, Makerere University, P.O. Box 7072 Kampala, Uganda; Centre for Health Professions Education, Stellenbosch University, Cape Town, South Africa

**Keywords:** Problem based learning, Feedback, Facilitator guide

## Abstract

**Background:**

Feedback delivery within a Problem Based Learning tutorial is a key activity for facilitators in order to enhance student learning. The purpose of this study was to explore students’ experiences of feedback delivery in a PBL tutorial and use this information to design a feasible facilitator feedback delivery guide.

**Methods:**

It was an exploratory qualitative study in which individual interviews and focus group discussions were conducted with students who had an experience of the tutorial process. Data were collected through audio recording and writing of field notes. Thematic analysis was employed to generate the reported themes.

**Results:**

Students suggested that facilitators need to give comprehensive feedback on their knowledge construction process as well as feedback on other generic skills outside the knowledge domain such as their communication skills within the tutorial, their participation and team work as well as their interpersonal skills and self-evaluation abilities. From the findings, a structured facilitator feedback delivery guide was developed.

**Conclusion:**

In this study, we propose a structured feedback delivery guide for PBL facilitators that captures not only knowledge, but also other generic competencies. The guide is feasible in a wide range of contexts where PBL is institutionalized.

## Background

Following its initial adoption in medical education at McMaster Medical School [1], Problem Based Learning (PBL) has been adopted across many medical schools [2–6]. The focus of PBL is the tutorial group which is comprised of a small manageable number of students being guided by a facilitator. During the tutorial process, students are presented with a learning problem which they are required to solve. The problem can take on many forms, for example clinical case scenario, patient narratives, medical images etc., all of which are the triggers of student discussion [7, 8]. The facilitator guides the discussion, ensuring that students are in line with the intended learning outcomes [9].

A key activity that occurs during a PBL tutorial is feedback delivery by the facilitator to the students. This feedback appraises student performance in relation to what was intended to be learnt. Feedback has been defined as information given to learners that assists them to identify strengths and areas of weakness that need attention [10, 11]. Feedback within a PBL tutorial is verbal and formative.

Formative feedback is intended to identify students’ strengths and learning gaps that need improvement [12, 13]. Such feedback is non-evaluative, timely, specific, and supports the learning process [14]. Published research emphasizes that feedback significantly promotes learning if delivered effectively [12, 14]. In the feedback process, one can recognize that there is an initial task to be performed, response is given about performance and a reaction occurs. In order for this reaction to occur, which may be positive or negative, students need to be given enough time to conceptualize the feedback received. Feedback enables students restructure their understanding and develop new knowledge constructs and it should therefore be integrated in routine teaching and learning activities [13, 14].

Previous literature has demonstrated that sometimes students receive differing feedback messages from facilitators [4], and students across tutorial groups often compare feedback received. Some facilitators may deliver feedback on only students’ knowledge while others may deliver feedback on aspects like communication in addition to knowledge. There is also a challenge of content expert facilitators versus non content expert facilitators. Content experts tend to give elaborate feedback on knowledge acquisition while non-content experts may not do this [15]. Other factors that could influence feedback utilization include overloaded and unfocused feedback [16–18]. There is therefore need to have a comprehensive feedback guide for PBL facilitators so that feedback delivered is focused on fairly similar pivotal domains across different tutorial groups.

### Context of the study

At Makerere University, College of Health Sciences (MaKCHS) where this study was conducted, the PBL curriculum was introduced in 2003/2004 academic year across all undergraduate health sciences programmes [19]. The curriculum is based on the SPICES model (i.e. Student-centered, Problem based, Integrated, Community based, Elective based and Systematic [20]. In this curriculum, learning is organized into specific courses, each being stratified into learning outcomes, content, learning methods, and assessment strategies. Each course commences with an over-view lecture by a content expert highlighting the scope and ensuring that learning outcomes are clear to the students. Thereafter, there are a variety of learning methods to engage the students which include; PBL tutorial sessions, clinical exposure, lectures, laboratory sessions, skills training and seminars. In each of these learning strategies, feedback is an essential element. However, this study focused on feedback within the PBL tutorial.

Each tutorial group is comprised of 10–12 students with a facilitator. One of the students takes on the role of group Chairperson to steer the discussion. Another student takes on the role of Scribe to write down key concepts being discussed. The group is principally managed by the students with the facilitator only guiding the learning process including delivering feedback.

Every group meets twice a week to discuss a learning problem. In the first meeting, students convene with their facilitator to handle a new problem which involves brainstorming, raising learning issues and discussing the problem in relation to prior knowledge. Learning gaps are then identified which become the learning objectives for self-directed study. At the second meeting (usually after 3 days), students and their facilitator re-convene to address the previous learning objectives. At each of the discussions, students receive feedback from the facilitator regarding performance.

### Aim of the study

The purpose of this study therefore was two-fold. First was to explore students’ experiences of feedback during PBL tutorials, and then utilize the students’ experiences to develop a structured facilitator feedback delivery guide. Our hope was that if tried out, the feedback guide can fairly standardize feedback delivery as it is likely to guide PBL facilitators on what key domains to concentrate when framing their feedback. Many recent reported accounts on feedback delivery have been from the perspective of faculty and feedback experts [16–18]. In this study, we set out to get the views of the recipients of that feedback-the students. Throughout this paper, the word ‘facilitator’ has been used synonymously with the word ‘tutor’.

## Methods

### Study setting

The study was conducted at Makerere University College of Health Sciences, Uganda.

### Study design

It was an exploratory qualitative study that utilized individual interviews and focus group discussions to explore students’ experiences and opinions of feedback in a PBL tutorial group.

### Study participants and sampling

Participants included third year undergraduate health science students from across five disciplines namely: medicine, dentistry, radiography, pharmacy and nursing. Third year students were included because they had previous experience of the PBL tutorial process and would thus be in a better position to share their experiences. Fifteen (15) interviews and five (5) focus groups were conducted. Each focus group had eight (8) students. Students for the interviews and focus group discussions were selected using purposive convenience sampling (i.e. those students who met the inclusion criteria and were readily available to the researchers). This method of selecting participants is common practice in qualitative research [21]. The final number of interviews and focus groups was determined at the point of data saturation where responses had become repetitive. Students who participated in the individual interviews did not participate in the focus group discussions. This allowed a wide range of students to participate resulting into a variety of experiences and opinions which added richness to the study. Interviews were conducted first before the focus group discussions.

### Data collection and management

Individual interviews and focus group discussions were used to collect data by the researchers. This was aimed at triangulating data collected, a technique that is widely applied in qualitative research [22]. While individual interviews generated significant responses, focus groups allowed collection of more detail on the subject as views from different members in the same group triggered more ideas for discussion which would otherwise have been forgotten [22, 23]. The interviews and focus group discussions were conducted in English. The questions for the interviews and focus group discussions were open-ended and semi-structured and generally explored the same subject. The questions sought students’ experiences and opinions of feedback received during PBL tutorials, the aspects of feedback the facilitators concentrated on and how they wanted feedback to be improved. These questions were constructed by the researchers based on literature and were first piloted to ensure that the correct information was collected.

A quiet room was chosen for the interviews and focus group discussions. Responses from participants were audio-recorded and later transcribed. Side field notes were also taken for purposes of reference. Data collected was put into electronic format and stored on one computer secured by a password and only accessible to the researchers.

### Data analysis

Thematic analysis was used. Raw data was read and through a series of iterative and inductive open and axial coding, codes were developed. Open coding involved reading through the transcribed raw data and identifying similar response phrases which became the codes [24]. Axial coding involved clustering codes of similar meaning into broader patterns and relating them to the original data, subsequently resulting into broader patterns called categories [22]. The categories were also related to each other and to the raw data and subsequently grouped into bigger themes which were used to report findings. Coding was manually carried out by the researchers. An inductive approach was adopted because it allowed constant comparison and synthesis of data being collected which ensured that themes naturally emerged from the data collected, a common practice in qualitative research [24, 25].

### Quality assurance

Participants were contacted to validate the emerging themes as a true representation of their responses. Researcher bias was minimized by having more than one researcher and the researchers reflecting and weaning off all pre-conceived ideas, assumptions or experiences on the subject under investigation which could have compromised findings (i.e. practicing reflexivity and bracketing).

### Ethical considerations

Participants provided written informed consent. No participant was identified by name and the responses were kept anonymous and confidential. Permission to conduct the study was granted by the Health Research Ethics Committee, Faculty of Medicine and Health Sciences, Stellenbosch University as well as the Research Ethics Committee, School of Medicine, Makerere University.

## Results

This study sought to explore students’ experiences and opinions of feedback in a PBL tutorial and use this information to develop a feasible feedback guide for PBL tutorial facilitators. These have been broadly categorized into two themes namely: feedback on knowledge; and feedback on generic skills. These key themes and focus areas under each theme have been summarized in Table 1.

**Table 1:** Showing the two major themes and respective focus areas for improvement

Major theme	Key focus areas for improvement
Feedback on knowledge	• Explicit feedback on understanding and discussion of key concepts • Feedback on level of prior knowledge • Need for feedback on how prior knowledge has been related to discussion • Feedback regarding learning gaps • Feedback on knowledge acquisition process
Feedback on generic skills	• Participation in discussion • Communication and interpersonal skills • Team work and collaborative learning • Time management • Leadership and management skills • Reflective ability

### Theme 1: Feedback on knowledge

Students who participated in this study suggested that the facilitator should give comprehensive feedback on knowledge of concepts in the problem presented. There was a common thread in the responses that facilitator feedback on knowledge was too narrow. Additionally, such feedback only addressed the issue of whether students had achieved the intended learning objectives. Suggestions were identified that facilitators need to explicitly give feedback on certain key areas. These included: student grasp and understanding of key concepts, level of prior knowledge and learning gaps that need to be addressed. The following responses were typical.

“*Most tutors only tell you whether you have derived the intended learning objectives or not…it would be good if we got feedback from different angles such as how good our prior knowledge was regarding the problem.*”

“*I noted that the PBL tutors ignore giving us feedback on some aspects such as our initial understanding of technical issues in the problem and how well we have discussed them…..I would advise that facilitators also give us feedback regarding our understanding the concepts in the problem.*”

From the responses above, it can be observed that although the facilitator delivered feedback regarding students’ acquisition of knowledge, the feedback was somewhat narrow and focused mainly on whether students had achieved the intended learning objectives. It appears students wanted a little more elaborate feedback on the knowledge acquisition process involving how well they related to their prior knowledge and level of understanding of key concepts in the problem.

### Theme 2: Feedback on non-cognitive generic skills

Many facilitators either did not give or gave very limited feedback regarding other non-cognitive skills outside the knowledge domain. Such non-cognitive attributes that did not feature much in the facilitator feedback included effective communication skills, participation, team work, collaborative learning, reflection, time management, maintenance of group dynamics and interpersonal skills.

“*During our orientation to PBL, we were told that besides content knowledge, we shall learn other aspects like communication skills and working as a team in our tutorials. However, none of my tutors has given me feedback regarding these within the tutorial.*”

“*Much as were assured that a PBL tutorial is an avenue for learning other skills like time management and collaborative learning besides knowledge, our tutors give us feedback on only knowledge gaps. I do not know how am fairing in those other skills.*”

“*I have come to appreciate that in a PBL group, I can learn how to discuss with colleagues, how to relate to people with different opinions and I have done my best to practice these. However, I do not know whether am well or need to improve….my tutor never mentions these softer skills apart from the hardcore medical content.*”

“*I think the tutor would have been good if he had given feedback regarding our initial prior knowledge and how we related it the tutorial problem we were discussing. This at least motivates me.*”

It is thus evident that feedback on other domains besides knowledge was lacking, yet these are emphasized within a PBL tutorial learning environment. In this study, it was also observed that facilitators did not deliver feedback on similar pivotal domains across different tutorial groups. Students said that some facilitators would talk about time management in the tutorial while others did not. Some facilitators would give them feedback regarding their participation while other would not.

“*We normally change facilitators after 5 weeks. I have noted that facilitators give feedback on different aspects…for example one can tell you about your communication while another will not. Tutors should follow the same procedure.*”

“*Our tutors do not give feedback following similar lines. Even on knowledge acquisition, different tutors will give feedback on different aspects…while some talk about how good or bad your prior knowledge was, others will never mention anything to do with prior knowledge. This needs to be improved.*”

### The PBL tutorial facilitator feedback guide

Following review of the student responses, a feedback delivery guide was developed aimed at ensuring that PBL facilitators deliver feedback on the same competency domains (Table 2). This guide can probably act as a resource for both expert and non-expert PBL facilitators. It emphasizes only those key learning aspects that the PBL tutorial aims at addressing. The guide outlines five key feedback domains against each of which are descriptors. The key feedback domains identified include: problem conceptualization and knowledge construction; participation and team work (collaborative learning); communication and interpersonal skills; time management and leadership; and reflective practice. It is these feedback domains that address most competencies meant to be learnt within an active PBL tutorial. Against each feedback domain is a list of questions that can guide the facilitator to frame his/her feedback.

**Table 2:** Structured feedback delivery guide for PBL tutorial facilitators

Feedback domain	Description
Problem conceptualization & Knowledge construction process	To what extent did students. • Understand the problem presented? E.g. understanding the central theme of the problem • Identify key concepts in the problem? • Activate their prior knowledge to solve the problem? • Formulate an action learning plan? E.g. deriving the learning objectives to cover knowledge gaps • Apply knowledge gained from self-directed learning? E.g. showing evidence of new acquired knowledge in the discussion • Use evidence gathered? E.g. supporting the discussion with opinions and ideas with reference to sources of information.
Participation and team work (Collaborative learning)	To what extent did students. • Share knowledge acquired? E.g. actively giving information in the discussion, clarifying issues and providing counter-arguments. • Ask questions for clarification of ideas and concepts within the group? • Clarify issues to peers? • Practice collaborative learning? E.g. willingness to learn from each other in a collaborative and not competitive manner.
Communication and interpersonal skills	To what extent did students. • Listen to each other during the discussion? • Express their ideas/arguments loudly, clearly, confidently and precisely? • Organize their ideas? E.g. demonstrating coherent and logical flow of ideas in their discussion/arguments. • Show respect, maturity, self-control and concern when discussing with colleagues in the group? • Demonstrate conflict resolution skills? E.g. addressing any conflict situations in a positive way where each member benefits resulting into shared learning.
Time management and leadership	To what extent did students. • Show punctuality? E.g. being in time for the tutorial. • Manage time schedules? E.g. addressing learning objectives in time, adhering to stipulated time in the tutorial discussion, remaining focused to issues. • Exercise leadership skills? E.g. the Student Chairperson controlling the group effectively, his/her strengths & weaknesses and what needs be improved.
Self and peer evaluation (Reflective practice)	To what extent did students. • Identify what they did well? • Identify gaps that need improvement? • Evaluate each other (peer to peer evaluation)? • Positively respond to feedback given?

N.B: The PBL facilitator should package his/her feedback message around the bulleted points against each domain. This feedback delivery guide is a supplement to the facilitator guide which has the detailed subject content, and not a substitute

## Discussion

### Students’ experiences and opinions of feedback in a PBL tutorial

This study sought to explore students’ experiences and opinions of feedback within a PBL tutorial. Using interviews and focus groups allowed a comprehensive exploration of these experiences. These included: the need for facilitators to give comprehensive feedback on the knowledge acquisition process that involves commenting on grasp of key concepts, use of prior knowledge and identification of learning gaps. Students also suggested the need to receive feedback on non-cognitive skills besides knowledge such as effective communication, adherence to ground rules established by the group and maintenance of group dynamics [3]. From the findings, it appears like PBL facilitators concentrated on informing students whether they had correctly identified the intended learning objectives in the hope that this will assist the students to learn. Whilst giving feedback regarding intended learning outcomes is a good idea, there is need to probably move beyond this.

Facilitators need to view the knowledge construction process during a PBL tutorial comprehensively and deliver feedback on various aspects involved in this process. For example, it would be good to inform students how well their prior knowledge was, how it linked with their discussion, how they interpreted or misinterpreted the concepts in the problem, how well they identified the learning gaps and how well they connected all this to their derived learning objectives. They can then be informed how their learning objectives compared with the intended institutional learning objectives. This comprehensive feedback on knowledge construction is likely to be useful in guiding students’ learning, an observation that has been previously reported [15].

Feedback on key non-cognitive skills was generally lacking. Non-cognitive generic skills refer to those soft skills besides knowledge that can be applied along with knowledge to perform a task [20]. These include: communication, interpersonal relations, conflict resolution, self-evaluation/reflection, team work, collaborative learning, reflective practice (i.e. thinking about one’s own learning processes). The importance of such skills for health professionals has been emphasized in PBL literature [9]. In traditional didactic teacher-centered learning, it was a challenge imparting these skills [6]. Literature on PBL is replete with documented accounts of the superiority of PBL tutorials over the more traditional pedagogical methods in its ability to provide learners with an opportunity to acquire non-cognitive skills besides knowledge [2–6]. Our findings are in agreement with this previous literature.

Based on findings from this study, which also resonate with previous studies, we advise PBL tutors to pay attention to not only knowledge, but also the other non-cognitive skills and deliver feedback to students about their performance as far as these skills are concerned. All these skills are evident within a PBL tutorial setting, though tutors sometimes tend to neglect them. If comprehensive feedback was framed around all these aspects of which knowledge is just part, students are more likely to be in a better position to come out as all-round professionals [15].

### The PBL tutorial facilitator feedback guide

Based on the observations from this study, we propose a feedback delivery guide that can assist tutors to frame comprehensive feedback within a PBL tutorial setting (Table 2). In developing the facilitator feedback guide, we were cognizant of time limitations tutors have within PBL tutorials and thus we made it highly structured by identifying key feedback domains and providing guiding questions to help the tutor frame his/her feedback.

Making it highly structured achieves three things: 1) it should be feasible and simple to implement and follow as it explicitly provides tutors with only those key areas to follow when formulating their feedback; 2) the guide could be one avenue through which students across different tutorial groups receive feedback on the same range of key competencies within a PBL group. Thus the challenge of having differing feedback messages or facilitators unknowingly neglecting some domains is probably addressed; and 3) the guide may support tutors to deliver high quality feedback that targets institutional learning outcomes. A key advantage of the guide is that it enables tutors to deliver feedback across all competencies acquired within a PBL tutorial. One can observe that the guide has not only knowledge, but also other competencies that need facilitator attention too.

Furthermore, in developing this guide, we envisaged that facilitators can probably frame their feedback around the same pivotal domains across different tutorial groups. It should be noted however, that this guide is for only the process of delivering feedback within the tutorial. It focuses on delivering feedback across similar domains by different facilitators within the different tutorial groups at a meta-level. The guide does not prescribe that facilitators should use exactly the same feedback language at content level. The guide may be applicable in a wide range of environments where PBL tutorials are institutionalized and each institution can customize the guide depending on the prevailing contextual factors.

The strength of our findings lies on the method used. Exploratory interviews and focus group discussions generated rich contextual responses from students. Additionally, the use of students, who are the recipients of feedback, was also a strength. This is because many feedback guidelines have relied on inputs and experiences from faculty and feedback experts.

The non-probability sampling and small participant numbers are limitations of this study. Involving other participants such as PBL facilitators, feedback experts and experts in group dynamics would probably have added more breadth to this study. Nonetheless, the study yields key insights on to which other studies can build. Although, we developed the feedback guide from this study which may be applicable across many settings, it probably needs further scrutiny. We thus encourage further research focusing on the application of this guide across different settings.

## Conclusion

This study explored students’ experiences and opinions of feedback in a PBL tutorial. The study has demonstrated that PBL facilitators need to provide comprehensive feedback on the knowledge construction process as well as give feedback on other non-cognitive skills outside the knowledge domain including effective communication, adherence to ground rules and maintenance of group dynamics. Subsequently, a feedback guide for PBL tutorial facilitators has been designed which is structured, feasible and applicable across a wide range of contexts.

## Abbreviations

PBL: Problem Based Learning
MaKCHS: Makerere University, College of Health Sciences
